# Longitudinal analysis of Socioecological obesogenic factors in a National Sample of U.S. children

**DOI:** 10.1186/s13690-020-00494-z

**Published:** 2020-11-13

**Authors:** TaeEung Kim, Junhye Kwon, Chung Gun Lee, Chang-Yong Jang

**Affiliations:** 1 Department of Epidemiology, University of California, Irvine, CA 92697 USA; 2 Department of Physical Education, College of Education, Seoul National University, 1 Gwanak-ro, Gwanak-gu, Seoul, 08826 South Korea; 3 Korea Institute of Sport Science, 727, Hwarang-ro, Nowon-gu, Seoul, 01794 South Korea

**Keywords:** Longitudinal design, BMI, Childhood obesity, Socioecological framework

## Abstract

**Background:**

Childhood obesity is a serious public health threat. Although many researchers conducted research on socioecological determinants of childhood obesity, their longitudinal effects remain inconclusive especially among young children. This study examined socioecological factors and associated transitions of children’s body mass index (BMI) status throughout children’s kindergarten to elementary school years, using data from a national longitudinal sample.

**Methods:**

The baseline sample of this study included 1264 children (weighted *N* = 379,297) extracted from the Early Childhood Longitudinal Study (baseline mean age: 5.24 years). The socioecological framework guided selection of socioecological obesogenic variables (e.g., family activity and parental involvement). Longitudinal ordered logistic regressions were performed to determine the associations between socioecological obesogenic variables and unhealthy/healthy changes in BMI status that captured transitions between healthy and unhealthy weight status (i.e., overweight, obesity, and severe obesity).

**Results:**

Children with Hispanic ethnicity and nonwhite, less socioeconomic and environmental support, and living in households with fewer family members were more likely than their counterparts to have unhealthy BMI status changes over time (all *p*s < 0.05). Over the study period, girls were less likely than boys to experience transitions to unhealthy BMI status (all *p*s < 0.05).

**Conclusion:**

As hypothesized a priori, the findings of the current affirmed multiple dimensions of how sociological obesogenic factors may influence children’s BMI status changes in a longitudinal setting. In order to maintain children’s long-term healthy weight, more attention should be paid to socioeconomic obesogenic factors surrounding children as well as individual determinants of obesity (e.g., being physically active and having well-balanced nutrition).

## Background

The overall prevalence of childhood obesity has demonstrated a slight decrease and leveling off since 2000 in the U.S. [1]. However, rates of severe obesity are rapidly increasing [2]. Approximately 4–6% of US children are severely obese [2, 3]. Severe obesity in children has recently received attention due to the serious exacerbation of other health issues (e.g., obstructive sleep apnea syndrome, eating disorders, nonalcoholic fatty liver disease, musculoskeletal problems) when compared to being overweight or obese [4].

Overweight designation for a child is defined as a body mass index (BMI) between the 85th and 95th percentiles for age and gender; obesity is defined as a BMI greater than the 95th percentile [5]; and severe obesity is defined as a BMI greater than the 99th percentile [4, 6]. Previous research [7, 8] have examined similar classification structures among children and adolescents.

Severe obesity is an urgent condition resulting in the need for immediate action, such as surgery, to prevent critical issues (i.e., death, disability). While previous studies have recognized the seriousness of severe obesity, research is still insufficient to recognize obesogenic factors in children. Unfortunately, obesity-related interventions that have been tried in children with severe obesity have been shown to be less effective (or the effectiveness was indecisive) compared to that of interventions in overweight and/or obese children and adolescents [9–11].

Most research on childhood obesity has focused on prevalence estimates at one time point. However, utilization of this paradigm provides little insight into new overweight, obese, or severely obese cases, including transitions between classifications (i.e., from normal to overweight). Simple obesity prevalence research is a limited account of the complex mechanisms associated with childhood obesity. This limited accounting can have profound practical implications for policymakers and practitioners.

Childhood is a critical period in human development [12]. During this time, individuals typically experience radical changes in physical activity and/or eating behavior patterns, resulting in significant changes in height and weight [13]. No studies to date have examined transitions of BMI status from preschool to elementary-school periods in young children, while controlling for childhood obesogenic factors. Given the inadequate amount of research on the subject, the current study was to examine the cause of the shift and/or maintenance of children’s weight from a socioecological obesogenic perspective.

## Methods

### Data sources

Data were obtained from the Early Childhood Longitudinal Study (ECLS-K), which followed children from kindergarten (1998–99 school year) through the 2007 school year, when most children were expected to be in eighth grade. The 1998–99 class cohort was a nationally representative sample of kindergartners, parents, teachers, and schools from across the US. The Institutional Review Board at Indiana University verified the current study as a non-human subject study. Participants included in data analyses represent all waves of data collection time points (i.e., school years 1998–99, 1999–2000, 2001–02, 2003–04, and 2006–07). Dropouts and subjects added to the sample at any time are not included in the current study.

Conceptual Framework.

The modified socioecological framework [14] was used as the conceptual basis of the current study, as this model incorporates key transitions in children’s lives. Because child data was nested in parent, family, and environmental settings, a multilevel research approach was needed to account for the significant implications of the hierarchical structure [14]. This approach encompasses the primary needs of children, parents, families, and community members related to childhood development. The assessment framework consists of three parts: (a) individual needs (i.e., health, education, emotional/behavioral development, identity, family, social relationships, social presentation, and self-care skills); (b) parenting capacity (i.e., basic care, ensuring safety, emotional warmth, stimulation, guidance, boundaries, and stability); and (c) family/environmental factors (i.e., community resources, family’s social integration, income, employment, housing, extended family, and family history and functioning).

### Measures

#### Outcome variables

BMI percentile was the main outcome variable in this study, which was calculated and categorized as follows. First, BMI was calculated as weight in kilograms divided by square of height in meters and considered in relation to age and gender [15] Then, BMI percentile was used to determine severity of childhood obesity. Four levels of childhood obesity were categorized in this study: normal weight (BMI 5th–85th percentile); overweight (BMI 85th–95th percentile); obesity (BMI equal to or greater than the 95th percentile); and severe obesity (BMI equal to or greater than the 99th percentile) [4, 6, 8].

The secondary goal of the current study was to examine the role of socioecological obesogenic factors when children transition from normal/healthy weight to being overweight, obese, or severely obese. From the main outcome variable of BMI, transitions among the four main levels of a child’s weight severity were recorded on a 7-point scale (from − 3 to 3). In this scale, 3 was considered “worsening,” recording three levels of worsening of BMI status from Wave 1 to Wave 2 (e.g., normal at Wave 1 to severely obese at Wave 2). Conversely, − 3 was considered “improving,” meaning that a child improved BMI from Wave 1 to Wave 2 (e.g., severe obesity at Wave 1 and normal body weight at Wave 2). A score of 0 represented no change in BMI between the two waves.

The objectives of this study were based on similar studies that have identified the relevant obesogenic independent variables and examined how socioecological factors influence BMI status and transitions in children. Thus, the same independent variables were employed.

#### Independent variables

Individual obesogenic variables included gender, ethnicity, age, amount of computer usage, and number of hours watching TV after dinner. Ethnicity was categorized into four groups: Hispanic, non-Hispanic white, non-Hispanic black, and other. The amount of computer usage was indicated by the number of times per week measured on a 4-point scale from 1 (*Never*) to 4 (*Daily*). The number of hours watching TV after dinner was assessed by the average number of hours spent watching TV or videos at home each day following the final meal of the day (range = 0–7 h).

Parental variables related to childhood obesity consisted of parental educational levels, mother’s weekly working hours, and parental involvement. The highest level of education of the head of household was assessed on a 9-point scale from 1 (*8th grade or below*) to 9 (*Doctorate or professional degree*). Mother’s working hours were measured by current employment status on a 4-point scale from 1 (*Not in the labor force*) to 4 (*35 h or more per week*).

Family functioning consisted of a single-parent variable, number of family members less than 18 years old, child’s primary caregiver, a poverty indicator, income level, TV restrictions at home, and food security. There were five categories of combined family structure associated with a child’s parent(s), which were restructured as a binary variable (e.g., two-parent family vs. other family structure). Previous studies have shown that the health status of children with two parents may differ from that of children who are from different family structures [16, 17]. Additionally, a 7-point scale for types of primary care was utilized, and restructured as a binary variable, with “0” representing non-parental care and “1” representing parental care, as children with parental care may show different health outcomes compared to those who are cared for by someone else [18–21]. Number of family members under 18 and household size were measured as a range of 1–11 and 2–17, respectively. Nine classification categories for the child’s primary caregiver living in the household were measured, and restructured as binary variables, including biological mother and father vs. other, because children cared for by biological parents may show different health outcomes [21–23] According to the US Census Bureau [24], a binary variable for poverty threshold was assessed in accordance with family size and number of children. Level of income was measured on a 12-point scale from *$5000 or less* to *$200,001 or more*. Additionally, the level of income was categorized into quintile indicators to secure an unbiased measurement of income level. To measure TV watching restriction for children, a binary variable for a family’s rule regarding TV was assessed. Finally, food security in the household was measured on a 4-point scale from *food insecure with hunger* to *food secure*.

One of the key environmental obesogenic factors in childhood obesity is school [25]. School environmental factors consist of three main variables: type of school, proportion of minority students, and proportion of students eligible for free or reduced lunch. The percentage of minority students was assessed on a 5-point scale from 1 (*Less than 10%*) to 5 (*75% or more*). To measure the proportion of students eligible for reduced lunch, a 5-point scale was used to assess percentage of student eligibility from 1 (*Less than 1%*) to 5 (*25% or more*). Lastly, urbanity level of where children live was measured as an obesogenic factor. The characteristics of children’s geographical residence were divided into three categories of urbanity: large city, mid-size city, and small town/rural area of a large city.

### Statistical analyses

Unweighted and weighted descriptive statistics of the sample were determined to describe demographic characteristics, such as age, gender, ethnicity, and BMI status, in terms of means, standard deviations, or percentages. Pearson’s *χ*^*2*^ tests and *t*-tests with weighted counts and column percentages were used to compare descriptive statistics. Two longitudinal ordered logistic regressions were used to examine associations between sociodemographic variables (e.g., family activities, parents’ health behavior), obesity status, and transition across all waves, respectively. First, a longitudinal ordered regression was performed to examine the associations between BMI status (i.e., normal, overweight, obese, and severely obese) when controlling for sociodemographic variables. Further, a longitudinal ordered regression (i.e., recovery, maintained weight, and worse BMI changes), which is a panel regression with a random effect, was performed to capture factors influencing children’s weight transitions across time waves, controlling for demographics, physical activity-related behaviors, and familial/environmental conditions. Stata® 15.0 and SAS version 9.4 were utilized for all statistical procedures, with a .05 alpha level and a 95% confidence interval.

## Results

### Descriptive statistics

Table 1 shows the baseline descriptive statistics of the study sample: unweighted (e.g., number of participants) and weighted (e.g., mean, *SD*, and percentage). Data from 1264 children were examined for this study (mean age = 5.24 years; range = 2–13 years; 48.87% female). Racial makeup consisted of Non-Hispanic whites (75.59%), Hispanic/other (17.37%), and Non-Hispanic blacks (7.05%). Three independent variables associated with BMI status (gender, parental income, urbanity) were significantly different at baseline (see Table 1).

**Table 1:** Weighted descriptive statistics at baseline, Early Childhood Longitudinal Study, 1998-2007, US

%(***n***), Mean(SD)							
Variables		Normal	Overweight	Obese	Severely Obese	Total	***p***-value
		*n* = 855	*n* = 236	*n* = 141	*n* = 32	*n* = 1264	
		(67.64%)	(18.67%)	(11.16%)	(2.53%)	(100%)	
**Dependent variables**							
**Covariates**	**1. Individual**						
	Age	5.25 (0.45)	5.24 (0.46)	5.18 (.40)	5.22 (0.43)	5.24 (0.45)	.42
	Female	50.77% (436)	49.89% (114)	33.40% (57)	56.99% (17)	48.87% (634)	< .05*
	Ethnicity						.15
	Non-Hispanic white	78.07% (665)	70.42% (180)	67.49% (106)	74.08% (24)	75.59% (975)	
	Non-Hispanic black	7.13% (48)	6.15% (15)	6.33% (5)	15.30% (4)	7.05% (72)	
	Hispanic & Other	14.80% (142)	23.43% (41)	26.18% (30)	10.62% (4)	17.37% (217)	
	Computer usage	2.30 (0.80)	2.30 (0.92)	2.30 (0.86)	2.33 (0.98)	2.30 (0.83)	.90
	Hours watching TV after dinner	0.69 (0.73)	0.89 (0.87)	0.79 (0.73)	0.84 (0.79)	0.74 (0.76)	.22
	**2. Parenting capacity**						
	Parents’ education						.47
	Lower than high school	1.93% (20)	2.61% (6)	2.56% (4)	0% (0)	2.08% (30)	
	High school	19.91% (164)	18.68% (50)	25.31% (41)	29.51% (11)	20.48% (266)	
	Greater than high school	76.40% (661)	73.98% (173)	70.66% (95)	70.49% (21)	75.27% (950)	
	Mom’s employment	2.88 (1.25)	3.10 (1.32)	3.09 (1.23)	3.13 (1.23)	2.95 (1.26)	.30
	Parent income	8.69 (2.73)	8.42 (3.32)	7.99 (2.89)	7.38 (2.98)	8.53 (2.87)	< .05*
	**3. Family Function**						
	Family structure	82.88% (742)	76.28% (183)	77.26% (116)	70.54% (26)	80.94% (1067)	.20
	Family size with members less than 18 years old	2.42 (0.93)	2.36 (0.99)	2.28 (0.95)	2.28 (1.22)	2.39 (0.95)	.30
	Family TV restriction	86.63% (764)	85.03% (201)	84.54% (125)	96.35% (31)	86.33% (1121)	.66
	Primary caregiver	46.84% (402)	52.97% (123)	48.61% (78)	43.16% (14)	47.97% (617)	.39
	Food security	0.34 (1.37)	0.30 (1.23)	0.37 (1.31)	0.48 (2.20)	0.34 (1.37)	.74
	**4. School**						
	School’s ratio of minorities	1.97 (1.24)	1.91 (1.41)	2.01 (1.43)	1.84 (1.41)	1.96 (1.29)	.67
	School free lunch program	23.23 (21.85)	25.72 (22.85)	25.25 (21.57)	35.32 (27.93)	24.23 (22.19)	.21
	**5. Environment**						
	Urbanity						< .01**
	Large city	22.57% (215)	17.0% (46)	23.17% (34)	17.00% (5)	21.62% (300)	
	Mid-size city	50.72% (369)	40.07% (93)	37.43% (47)	41.23% (12)	47.34% (521)	
	Small town and rural	26.71% (271)	42.85% (97)	39.40% (60)	41.77% (15)	31.04% (443)	

*p < .05; **p < .01

*N* = 1264; weighted *N* = 379,297;

Most children showed even transitions (*n* = 1092; 87.71%) at baseline, followed by improved transitions (*n* = 103; 7.71%) and worse transitions (*n* = 69; 4.58%), respectively. Six independent variables associated with the seven levels of children’s weight transitions were significantly different at baseline (age, hours watching TV after dinner, food security, school minority status, school socioeconomic status [SES], urbanity; see Table 2).

**Table 2:** Weighted descriptive results of weight transitions at baseline, Early Childhood Longitudinal Study, 1998-2007, US

% (***n***), mean (SD)							
Variables		Improved^**a**^ (−2)	Improved^**a**^ (−1)	Even^**b**^ (0)	Worse^**c**^ (1)	Total	***p***-value
		*n* = 9	*n* = 94	*n* = 1092	*n* = 69	*n* = 1264	
		(.49%)	(7.22%)	(87.71%)	(4.58%)	(100%)	
**Dependent variables**							
**Covariates**	**1. Individual**						
	Age	5.22 (.53)	5.12 (.34)	5.25 (.45)	5.30 (.51)	5.24 (.45)	< .05*
	Female	44.42% (4)	47.73% (37)	49.36% (555)	41.77% (28)	48.87% (624)	.79
	Ethnicity						.16
	Non-Hispanic white	76.92% (6)	69.04% (76)	77.04% (847)	57.91% (46)	75.59% (975)	
	Non-Hispanic black	6.57% (1)	2.95% (3)	7.17% (60)	11.13% (8)	7.05% (72)	
	Hispanic & Others	16.52% (2)	28.02% (15)	15.79% (185)	30.96% (15)	17.37% (217)	
	Computer usage	2.00 (0.87)	2.26 (0.95)	2.30 (0.81)	2.37 (0.84)	2.30 (0.83)	.17
	Hours watching TV after dinner	0.67 (0.86)	0.76 (0.79)	0.72 (0.75)	1.03 (0.76)	0.74 (0.76)	< .01**
	**2. Parenting capacity**						
	Parents’ education						.66
	Lower than high school	10.42% (1)	.42% (2)	2.19% (26)	1.66% (1)	2.08% (30)	
	High school	16.90% (1)	15.23% (18)	20.75% (230)	23.87% (17)	20.48% (266)	
	Greater than high school	72.61% (7)	80.44% (72)	75.08% (821)	71.01% (50)	75.27% (950)	
	Mom’s employment	3.00 (1.39)	3.24 (1.08)	2.91 (1.26)	3.20 (1.37)	2.95 (1.26)	.11
	Parents’ income	8.78 (2.77)	8.48 (3.50)	8.58 (2.77)	7.81 (3.35)	8.53 (2.87)	.23
	**3. Family Function**						
	Family structure	93.38% (8)	77.80% (74)	81.55% (936)	72.79% (49)	80.94% (1067)	.50
	Family size with members less than 18 years old	2.56 (1.87)	2.34 (0.94)	2.40 (0.94)	2.25 (0.99)	2.39 (0.95)	.45
	Family TV restriction	100.00% (9)	80.74% (76)	86.77% (975)	85.29% (61)	86.33% (1121)	.65
	Primary caregiver	60.54% (6)	61.38% (51)	46.72% (520)	49.51% (40)	47.97% (617)	.38
	Food security	0	.44 (1.29)	.35 (1.39)	.12 (.63)	.34 (1.37)	< .01**
	**4. School**						
	School’s ratio of minorities	1.67 (0.74)	2.01 (1.32)	1.96 (1.27)	1.94 (1.59)	1.96 (1.29)	< .05*
	School free lunch program	12.36 (10.82)	25.47 (15.44)	23.88 (22.45)	29.61 (25.07)	24.23 (22.19)	< .01**
	**5. Environment**						
	Urbanity						< .01**
	Large city	0% (0)	21.58% (27)	22.14% (264)	14.05% (9)	21.62% (300)	
	Mid-size city	64.08% (6)	29.63% (30)	49.34% (463)	35.14% (22)	47.34% (521)	
	Small town and rural	35.92% (3)	48.79% (37)	28.52% (365)	50.82% (38)	31.04% (443)	

*p < .05; **p < .01

*N* = 1264; weighted *N* = 379,297

^a^ Any improved BMI status transition compared to each wave (−3, −2, and − 1)

^b^ Any unchanged BMI status transition compared to each wave (0)

^c^ Any worsened BMI status transition compared to each wave (1, 2, and 3)

### Distribution of children’s BMI status by year of study period

**Fig. 1:**
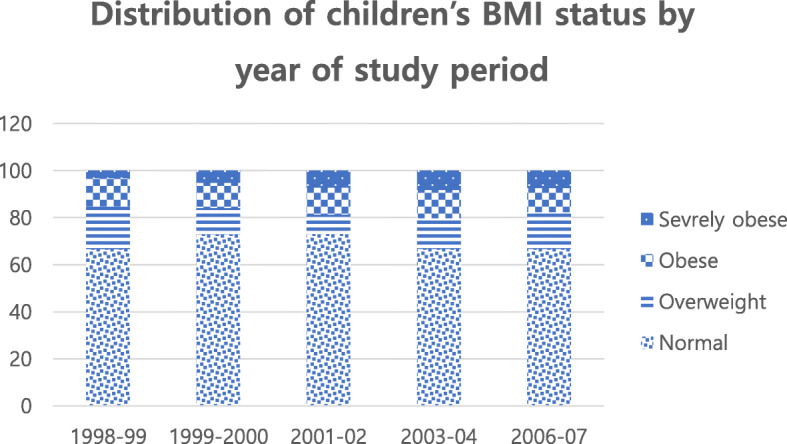
Distribution of children’s BMI status by year of study period, Early Childhood Longitudinal Study, 1998-2007, US

Fig. 1 illustrates the overall BMI status trajectories over the elementary years. Over time, the percentage of children with normal weight decreases while the proportion of obesity and severe obesity in children increases. Figure 1 shows only children’s BMI status trajectories by gender over the elementary years. Boys showed consistently higher BMI status levels than girls in each wave and boys had a significantly higher increase in BMI status level than girls over the elementary years (Fig. 1).

### Primary analysis of the relationship between socioecological factors and BMI status

Three obesogenic predictors were statistically significant (see Table 3). As age increased by one unit, the likelihood of experiencing weight gain increased over all periods (*OR* = 1.47, 95% CI = 1.20–1.79). Girls were less likely to experience weight gain compared to boys over time (*OR* = 0.16, 95% CI = 0.06–0.38). Further, Hispanic/other racial group were more likely to gain weight compared to non-Hispanic whites (*OR* = 4.34, 95% CI = 1.11–16.96).

**Table 3:** Weighted longitudinal ordered logistic analysis of random effects, Early Childhood Longitudinal Study, 1998-2007, US

Variables		Severity of Obesity		Transition of Children’s BMI	
		OR	CI	OR	CI
**Covariates**	**1. Individual**				
	Age	1.47**	1.20–1.79	0.85*	0.74–0.98
	Female	0.16**	0.06–0.38	0.71**	0.57–0.90
	Ethnicity				
	Non-Hispanic white	–	–	–	–
	Non-Hispanic black	0.13	0.01–13.15	1.10	0.66–1.83
	Hispanic and Others	4.34*	1.11–16.96	0.84	0.59–1.19
	Computer usage	0.96	0.68–1.36	0.93	0.79–1.11
	Hours watching TV after dinner	1.25	0.85–1.83	0.86	0.71–1.05
	**2. Parenting capacity**				
	Parents’ education				
	Lower than high school	–	–	–	–
	High school	0.08*	0.01–0.78	0.72	0.28–1.86
	Greater than high school	0.02**	0.01–0.22	0.79	0.31–2.03
	Mom’s employment	0.98	0.70–1.39	1.1	0.97–1.26
	Parents’ income	1.00	0.78–1.29	0.98	0.92–1.05
	**3. Family Function**				
	Family structure	0.09	0.01–1.19	0.99	0.69–1.43
	Family size with members less than 18 years old	0.53**	0.36–0.79	1.15	0.98–1.35
	Family TV restriction	0.77	0.36–1.68	0.99	0.64–1.54
	Primary caregiver	0.43	0.13–1.46	0.85	0.64–1.13
	Food security	0.85	0.72–1.25	1.02	0.82–1.26
	**4. School**				
	School’s ratio of minorities	1.86**	1.18–2.93	0.91	0.80–1.03
	School free lunch program	1.00	0.96–1.03	1.01	1.00–1.02
	**5. Environment**				
	Urbanity				
	Large city	–	–	–	–
	Mid-size city	0.77	0.32–1.86	0.82	0.58–1.15
	Small town and rural	5.35**	1.65–17.33	0.79	0.55–1.12

*p < .05; **p < .01

*N* = 1166; weighted *N* = 379,297

Only parental education level was statistically significant in predicting BMI status over time. The weight gain of the children whose parents graduated high school or graduated from higher than high school was significantly less (*OR* = 0.08, 95% CI = 0.01–0.78 and *OR* = 0.95, 95% CI = 0.00–0.22, respectively) than children whose parents did not graduate from high school. Additionally, as family size increased by one member under 18, the likelihood of weight gain also decreased (*OR* = 0.53, 95% CI = 0.36–0.79).

Two school and environmental factors (i.e., school’s ratio of minorities and urbanity) were statistically related to BMI status. Children in schools with a higher ratio of minorities were at a higher risk of experiencing weight gain (*OR* = 1.86, 95% CI = 1.18–2.93). Additionally, children living in the small towns and/or rural areas were more likely to gain weight (*OR* = 5.35, 95% CI = 1.65–7.90).

### Primary analysis of the relationship between socioecological factors and BMI transitions

Only two individual socioecological factors were statistically significant predictors of children’s weight transitions (see Table 3). As age increased by one unit, the likelihood of a weight-gain transition decreased (*OR* = 0.85, 95% CI = 0.74–0.98). Additionally, girls were less likely to experience weight-gain transitions compared to boys over time (*OR* = 0.71, 95% CI = 0.57–0.90).

## Discussion

This was the first longitudinal study to examine the association between sociodemographic variables and children’s BMI transition trajectories in a large sample of US children. The purpose of this study was to explain the cause of the shift and/or maintenance of children’s weight from a socioecological obesogenic perspective. Hispanic or non-white, socio-economic and environmentally insufficient children with fewer family members suffered more negative BMI transitions than those who did not. In addition, female children suffered more unhealthy BMI transitions than boys. The effects of ethnicity on obesity observed in this study were noteworthy, as children classified as Hispanic/other were more likely than non-Hispanic white children were to become overweight or obese. Previous research has shown that non-Hispanic black and Hispanic children tend to become more obese as they age [2, 26]. Unfortunately, few studies have been conducted comparing associations with ethnicity and obesity in children. Therefore, increased research should be performed on this topic to aid in the development of more effective obesity policies and interventions.

The current finding that parental educational attainment demonstrates an inverse relationship with child obesity is in line with previous research [27, 28], with effects likely due to the parent possessing more information/knowledge regarding overall child health and nutrition. Additionally, parents have a positive influence on childhood obesity when they praise children for making healthy choices [29].

Considering the young age of children in the current study, mother’s job status was hypothesized as having a significant impact on BMI. This is because care of young children in the home is often the responsibility of the mother [30]. However, results indicated that job status was not a significant factor in BMI. Rather, consistent with previous research by Gonzalez-Casanova et al. [31], having more family members under 18 in the household was associated with a lower likelihood of children gaining weight over time. It is believed that children with more families under the age of 18 will have more opportunities to participate in various physical activities due to more families, share more and better health knowledge and behavior, and stay healthier than children who are not mentally through mutual emotional and emotional stability and exchange with each other due to more families.

The current findings also show a positive association between the ratio of minority students in a child’s school and BMI. This is in line with previous studies, which have reported that Mexican and non-Hispanic black children are more likely to be overweight/obese, when compared with non-Hispanic white children [2, 26, 32].

The current study found that the level of urbanity played a key role in affecting obesity in the children. Specifically, children living in small towns showed significantly higher rates of negative BMI changes than children living in large cities. This may be because cities and urban areas provide more neighborhood amenities associated with BMI, such as sidewalks/walking paths, parks, playground areas, and recreation/community centers [33–36]. In addition to accessibility, the quality of facilities in large cities can also make a difference, because children can experience a variety of activities and have more chances for interaction [36–38]. Rural children also have relatively fewer opportunities to access fresh vegetables and various nutritious food ingredients than urban children, compared to children in metropolitan and urban areas [33, 34, 37, 38], and this is also an important determinant of child obesity. It is thought that the degree of suffix will indirectly affect these results.

Overall, findings indicated, as predicted, fewer individuals demonstrating no change in BMI percentile than demonstrating negative BMI transitions. This is consistent with a study by Ogden et al. [32], which examined the BMI levels of children and adolescents aged 2–19 years of age and found increased rates of children becoming overweight and/or obese over a 10-year period.

Compared to girls, more boys demonstrated negative BMI shifts over time in the current study, which mirrors results reported in previous studies examining childhood obesity in the US [32, 39]. In other words, as age increased, the proportion of children demonstrating negative weight-change transitions was higher for boys than for girls.

Cunningham, Kramer, and Narayan [40] examined obesity over a nine-year period in young children, and found that children between the ages of 5–14 demonstrated the greatest likelihood for obesity. Furthermore, children who were obese when entering kindergarten were more likely to remain obese throughout elementary school. The present results also indicated that children tended to remain obese or severely obese once their weight transitioned to overweight or obese. Children who returned to a normal weight or demonstrated positive weight change were relatively rare. These results are also similar to a previous study, which revealed that a large proportion of adolescents that experienced weight gain became or remained obese during the transition to adulthood during the five-year study period [41]. A separate longitudinal study examining severe child obesity [8] found that, while overall rates of overweight, obesity, and severe obesity tended to decrease, most of the normal weight and extremely obese children maintained their weight through the five-year study period. However, children who were initially overweight or obese tended to show weight gain during this period of time.

In general, a child’s age plays a negative role in changes in his/her BMI [1, 32]. With age, children are more likely to be exposed to poor environments and learn about unhealthy behaviors/habits from peers [25, 42]. This makes older children tend to demonstrate more unhealthy eating habits (e.g., consuming junk food, higher calorie intake, unbalanced nutrition) and a more sedentary lifestyle (e.g., computer use, watching TV) [38, 43]. Therefore, the effects of these factors on children’s weight should be carefully monitored.

Boys demonstrated more negative weight shifts over time than girls in the current study, which is a finding similar to other studies of gender differences in body weight changes [44, 45]. More specific and effective anti-obesity policies that consider gender differences in children should be implemented to reduce the gender disparities of childhood weight change.

Childhood is a critical period for the developing fundamental behaviors such as active and healthy eating habits (e.g., more being physically active, consuming more fresh fruits and vegetables, avoiding sugary food, eating low-fat foods and limiting junk food), which will contribute to the health of old age [11, 12, 18, 20, 21, 33]. In addition, it is a significant developmental stage that substantially affects healthy behavior throughout life the transition from adolescence to young adulthood. Unhealthy behaviors or practices adopted in the early stages of development will result in poor quality of life in later years [20, 21, 33]. If an individual adopts negative or adverse health behaviors or practices, these behaviors are more likely to persist and the individual may continue to deteriorate.

This study should be interpreted with the following limitations. First, this study utilized single-day, parent-reported measurements to assess all study variables, which may be inaccurate due to recall, respondent, or interview bias. Notably, there is a tendency for parent-reported overestimations and underestimations of weight for younger/low BMI and older/high BMI children, respectively [46, 47]. However, differences between measured and parent-reported body weight and height in this sample were small [47], and the longitudinal study design resulted in repeated measurements of children’s height and weight over time, reducing error [48, 49]. Second, the small sample size (e.g., Hispanic and others) observed may limit the generalizability of the current findings. A larger and more diverse sample could generate better results. Third, the dataset did not include measures of healthy eating behaviors, such as fruit/vegetable consumption, which have been both directly and indirectly associated with the prevalence of childhood obesity. Forth, although the data used in this study was collected quite some time ago, it is the most recent data we could use in a longitudinal setting. As the data is collected every 10 years, future studies should consider analyzing next period of the data. Finally, the contribution levels of only single effects were considered in the current study. Future studies should also examine whether multilevel effects can contribute to children’s BMI status over time.

## Conclusions

Despite the limitations discussed above, this research is an important study of the socioecological obesity factors that affect children’s BMI status transitions. Based on this research, it is necessary to develop and implement a long-term, fully developed, and systematic obesity policy. The implementation and implementation of child obesity intervention programs or policies is essential for health educators, experts, policy makers and stakeholders to improve the quality of life of children and adolescents. One of the most important reasons is that the cost-benefit ratio of medical or social intervention is most advantageous in childhood. Therefore, if policy makers want better outcomes for intervention policies in terms of cost-benefit ratios, they should target developmental stages in childhood and adolescence. In other words, compensation for medical or social intervention in adulthood is expected to be small.

## Data Availability

The datasets generated and/or analyzed during the current study are available in the [Early Childhood Longitudinal Study-Kindergarten cohort (ECLS-K)] repository, [https://nces.ed.gov/ecls/].

## Abbreviations

BMI: Body Mass Index
ECLS-K: Early Childhood Longitudinal Study-Kindergarten cohort
SES: Socioeconomic Status
STATA: Software for Statistics and Data Science
SAS: Statistical Analysis System
